# Voluntary modulation of mental effort investment: an fMRI study

**DOI:** 10.1038/s41598-017-17519-3

**Published:** 2017-12-08

**Authors:** Omar T. Khachouf, Gang Chen, Davide Duzzi, Carlo A. Porro, Giuseppe Pagnoni

**Affiliations:** 1 Department of Biomedical, Metabolic and Neural Sciences, University of Modena and Reggio Emilia, Modena, 41125 Italy; 2 Scientific and Statistical Computing Core, National Institute of Mental Health, National Institutes of Health, Bethesda, MD 20892 USA; 3 Center for Neuroscience and Neurotechnology, University of Modena and Reggio Emilia, Modena, 41125 Italy

## Abstract

Mental effort is a common phenomenological construct deeply linked to volition and self-control. While it is often assumed that the amount of exertion invested in a task can be voluntarily regulated, the neural bases of such faculty and its behavioural effects are yet insufficiently understood. In this study, we investigated how the instructions to execute a demanding cognitive task either “with maximum exertion” or “as relaxed as possible” affected performance and brain activity. The maximum exertion condition, compared to relaxed execution, was associated with speeded motor responses without an accuracy trade-off, and an amplification of both task-related activations in dorsal frontoparietal and cerebellar regions, and task-related deactivations in default mode network (DMN) areas. Furthermore, the visual cue to engage maximum effort triggered an anticipatory widespread increase of activity in attentional, sensory and executive regions, with its peak in the brain stem reticular activating system. Across individuals, this surge of activity in the brain stem, but also in medial wall cortical regions projecting to the adrenal medulla, positively correlated with increases in heart rate, suggesting that the intention to willfully modulate invested effort involves mechanisms related to catecholaminergic transmission and a suppression of DMN activity in favor of externally-directed attentional processes.

## Introduction

Mental effort is a concept commonly understood as a deployment of mental resources in a demanding task that needs to be willfully maintained, along with the feeling of exertion that may ensue. Its importance as a psychological construct is highlighted by the frequency with which it occurs in daily life language. The widespread use of exhortations to “make an effort” or to “put more effort” in a task, as well as the opposite suggestions of “not to force it” or “to relax” (more common in the context of artistic or expert performance), point to a phenomenological entity perceived as capable of influencing the quality and the outcome of an action in a variety of situations. Notably, the construct of mental effort is of such general import that its appeal cuts across several disciplinary domains: it is not only a concept of evident philosophical moment, with direct links to the “hard” questions of consciousness and free will, but also a dimension of cognition with important pedagogical^1–3^ and clinical implications^4,5^, potential for novel contributions to the neuroscience of executive function and decision-making^6–12^, and direct applicability to the field of ergonomics and human factors^13–15^. Nonetheless, the relationship among the willful application of mental effort, its subjective perception, and its effects on cognitive performance, as well as the neural circuitry underlying effort processing, remains a topic insufficiently understood to this date (but see Shenav *et al*.^16^, for an excellent overview of recent progresses).

In his seminal work, “Attention and effort”, Daniel Kahneman proposed the equivalence of the problem of mental effort to that of the allocation of limited attentional resources to a demanding task^17^. Notably, Kahneman considered the investment of effort in a task as a largely automatic process, dependent on task difficulty, rather than a mental factor that could be applied in a graded fashion by an act of will. While this approach had the great benefit of operationalizing effort, it also left the aspects of voluntary control and subjective perception of effort, along with their neural substrates, virtually unexamined. More recently, Dehaene *et al*.^18^ have proposed a theoretical model in which tasks that are performed effortlessly are characterized by a neural activation space limited to specialized modules of primary processing (such as perceptual, motor, and long-term memory processing); effortful tasks, on the other hand, require the ad hoc long-range integration and coordination of these primary modules, a costly process but one that also allows for cognitive flexibility. This more “global” computational workspace would be implemented by a distributed set of neurons with long axonal projections, such as the Von Economo neurons of the fronto-insular and anterior cingulate cortices^19^, with its overall state of activation under control of brain stem arousal circuits. Finally, the role of motivational processes has been highlighted in a recent model of attentional effort, described as “a motivated activation of attention systems in order to stabilize or recover attentional performance in response to the detection of errors and reward loss or, more generally, deteriorating attentional performance”^6^. According to this model, the prefrontal cortex (PFC) integrates motivational information from the dopaminergic mesolimbic reward system, and enhances signal processing in relevant sensory and associative posterior cortices via increased cholinergic transmission mediated by the basal forebrain. The deployment of more or less attentional effort is determined here by the magnitude of the reward associated with correct task performance, and thus the potential modulation of invested effort by a purely voluntary act is again not taken into consideration.

In line with the models discussed above, neuroimaging data have provided ample evidence for a general involvement of the dorsal aspect of the medial frontal cortex (MFC) in the context of effort. The anterior and mid-cingulate cortex (ACC, MCC) in particular, whose activity tends generally to increase with the difficulty of a task^20,21^, has been proposed as a key structure in cognitive control for the detection of performance errors^22,23^, for signalling response conflict^24–26^, and for encoding effort^27–29^, although its precise role is yet to be unambiguously determined^30,31^, perhaps because of its functional and structural heterogeneity^32^.

Mental effort may not be a sole matter of positive activation of attentional circuits but, at least in externally-directed tasks, may also involve the *de*-activation of a well-defined cortical network displaying high metabolic activity during wakeful rest^33^. Such “default mode network” (DMN), as it is commonly referred to^34^, has been implicated in the generation of spontaneous, task-unrelated thoughts^35–37^, whose occurrence represents a source of internal noise that can affect negatively the performance of a resource-intensive task^38,39^. The activity of the DMN has been in fact shown to fluctuate with an opposite phase with respect to the set of brain regions known to underlie the active engagement of attention to external stimuli^40^, suggesting a trade-off between the processing of internal and external sources of information mandated by the finiteness of cognitive resources^35^.

The present study sought to clarify a specific aspect of effort processing, namely the situation where the level of invested mental effort is determined by an act of will (*i.e*. endogenous), as compared to task-determined (*i.e*. mandated by the difficulty of the task, or exogenous). The relationship between these two instances of mental effort, and their neural bases, was examined using functional magnetic resonance imaging (fMRI) during a Stroop colour-word interference task. More specifically, we were interested in the following research questions: (*i*) what are the neural correlates of complying with the instruction to voluntarily modulate the degree of invested effort in a task; (*ii*) whether, and in which way, the voluntary modulation of invested effort affects performance, and (*iii*) whether the above effects depend on the intrinsic difficulty of the task. To this aim we employed a 2 × 2 repeated-measures experimental design, with endogenous and exogenous effort as independent factors. The levels of exogenous effort corresponded to the Stroop task’s congruent (easy = low-effort) and incongruent (difficult = high-effort) trial types. The degree of endogenous effort engagement was varied by instructing the volunteers to perform the Stroop task either “with maximum exertion”, or “as relaxed as possible”. We hypothesized that the instruction cue to invest maximum effort would elicit a transient activation of a widespread network of executive brain regions, corresponding to a generalized response of increased arousal and preparation for action. We also predicted that during the performance of the actual task blocks, when external cognitive load sharply increased, this initial difference in brain activation associated to high/low endogenous effort investment would become less conspicuous, but still be present and associated with performance changes.

## Methods

### Participants

A total of 55 right-handed volunteers, recruited mostly from the local college community, entered the study. Of these, only 45 participants who met the criterion of responding correctly to at least half of the incongruent trials in each run of the Stroop task (see Sec. *fMRI task*) were included in the final analysis. The included sample had an average (SD) age of 21.9 (2.2) years. Handedness was assessed by the Edinburgh inventory^41^. Participants were screened for compatibility with MR scanning and gave written informed consent for a protocol approved by the University of Modena and Modena Province Ethics Committee, Italy, in accordance with the Code of Ethics of the World Medical Association (Declarations of Helsinki of 1975) for experiments involving humans. Participation to the study was compensated with either a €20 sum or course credits.

### MRI session

MRI scanning was performed at the N.O.C.S.A.E. Hospital of Baggiovara (Modena, Italy), using a 3-Tesla Philips Achieva scanner. The session consisted of the acquisition of a whole-brain high-resolution T1-weighted image for anatomical reference (180 sagittal slices, 1 mm isotropic voxels), and six functional echo-planar imaging (EPI) runs (112 volumes/run, TR 2.5 s, 35 axial slices, 3 mm isotropic voxels), during which volunteers performed the task described in the following section.

### fMRI task

**Figure 1:**
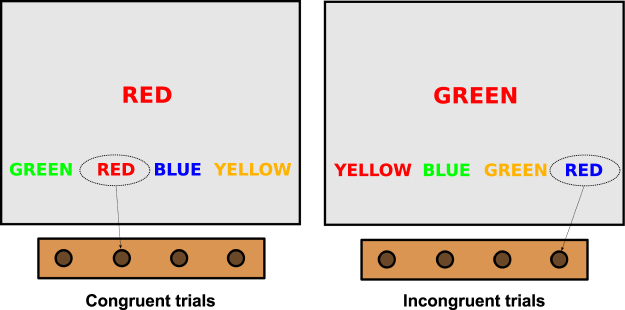
The Stroop task. Example screenshots for congruent and incongruent trials are shown on the left and the right side, respectively. The correct button presses are indicated by arrows. Note that the order of the labels at the bottom of the screen was randomly assigned at each trial, and that in the incongruent trials (but *not* in the congruent trials) the font color of such labels was also discordant with their text.

We used a 4-colour version of the Stroop task, with a finger-press response modality on a MRI-compatible button-box^42^. Participants were asked to attend to the visual stimuli presented on a MRI-compatible monitor and “press the button corresponding to the position of the bottom label whose *text* matches the *font colour* of the centrally-presented colour word” (Fig. 1). The “Stroop effect”^43^ consists in longer response times and greater number of errors for the *incongruent* trials (where the font colour of the centrally-presented word does not match its semantic value–*e.g*. the word “RED” in green fonts), compared to *congruent* trials (*e.g*. the word “RED” in red fonts).

Stimuli were arranged in a mixed block/event design. Each of the 6 EPI runs (≈5 min/run) included four 30-second task blocks (T) and five 25-second rest periods (R) of passive fixation of a centrally-presented cross, according to an RTRTRTRTR sequence; within each task block, 6 incongruent and 6 congruent trials were presented for 2 s each, in pseudo-random order with a 2.5 s intertrial interval (*i.e*. adjacent trials were separated by a 500-millisecond blank screen). Response times and errors were recorded in a log file by the stimulus-presentation computer, via the software PsychoPy (www.psychopy.org)^44^.

Crucially, for the aim of the present study, participants were asked to perform odd- and even-numbered runs with two different degrees of (endogenous) effort investment: (*a*) “with maximum exertion” (EXR condition), or (*b*) “as relaxed as possible” (RLX condition). These instructions were visually presented in the center of the screen at the beginning of each task block for 2 s (*cue* stimulus), so that participants would be prompted to engage either maximum or minimum effort just before task performance; they were also instructed to simply rest, with the gaze on the fixation cross, during the rest blocks separating the Stroop blocks. A first batch of participants (N = 27) performed odd-numbered runs (1, 3, 5) in the EXR condition and even-numbered runs (2, 4, 6) in the RLX condition; for a second batch of participants (N = 18), the order of conditions was reversed. To ensure that the instructions were fully understood, participants practiced the task on a laptop, under the experimenter’s supervision, before entering the MR scanner room. When debriefed after the practice runs, participants reported overall an adequate understanding of the difference between the two endogenous effort conditions.

### Subjective ratings and other measures

At the end of each EPI run, participants were asked to subjectively assess different aspects of the task workload with the NASA-TLX rating instrument^45^. The same button-box employed for the Stroop task was used to move a slider along six on-screen computerized visual-analog scales (VAS), corresponding to the following psychological dimensions: Mental Demand, Physical Demand, Temporal Demand, Performance, Effort, and Frustration Level (see Supplementary Information for details). All VAS marks were converted to 0–10 values for subsequent analyses; they were not transformed to weighted scores, as the actual usefulness of the latter procedure is controversial^46^. Heart rate was continuously recorded with a finger oxymeter during the MRI session.

### Data analysis

#### Effects of interest

Given the explanatory factors of endogenous effort condition (*cond*: EXR, RLX) and stimulus type (*stim*: incongruent, congruent), the primary contrasts of interests were: (*i*) EXR-RLX, representing the main effect of voluntary modulation of effort investment, and (*ii*) *cond* × *stim*, representing the interaction of effort investment and task difficulty or, in other words, the interaction of endogenous and exogenous effort recruitment. Since we observed a marked practice effect during runs 1 and 2 (both response times and number of errors decreased significantly), the data from these runs were discarded so that the final analyses for all types of data (behavioral, fMRI, heart rate, and subjective reports) included runs 3–6 only. Effect sizes were expressed in terms of the equivalent Pearson’s correlation coefficient, derived from the *t*-statistics using the formula $r={\rm{sign}}(t)\sqrt{{t}^{2}/({t}^{2}+{\rm{df}})}$, where *t* = *t*-value and df = degrees of freedom.

#### Behavioural data

A preliminary analysis of response times (RT) and number of errors in the Stroop task was first conducted within a mixed-model framework, using the package “nlme” of the statistical software R (www.R-project.org). As model searching showed negligible effects of the effort condition order across runs (1st batch vs 2nd batch of participants), of its interactions with the effects of interest, and of error type (omission, commission), a simplified analytical strategy was chosen. The average RT values and number of errors within each *cond* × *stim* cell were first computed for every participant, and these values were then analyzed at the group level with a factorial repeated-measures ANOVA (with *stim*, *cond*, and *cond* × *stim* as explanatory variables).

#### Oxymeter data

Heart rate (HR) values during task performance and rest (passive fixation) intervals were obtained with the following procedure. For each run, the oxymeter trace was first split up into task and rest blocks segments, which were bandpass-filtered with a 0.5–3.33 Hz frequency cutoff. The spectral density of each segment was then estimated by a smoothed periodogram with a 5-point modified Daniell filter. Finally, the periodogram’s dominant frequency (the frequency with maximum amplitude) was identified and chosen as the representative HR value during the selected block, followed by averaging across blocks and runs for each condition of endogenous effort investment (EXR, RLX). The analysis was performed using a factorial repeated-measures ANOVA, with HR as the dependent variable, and condition (EXR, RLX), state (task, rest), and their interaction as within-subject explanatory factors.

#### Correlations between behavioural, cardiac, and psychometric data

For exploratory purposes, we investigated the presence of across-subjects correlations between EXR-RLX differences in RT, HR values, and TLX-NASA ratings. Given the large number of correlations examined, we did not employ Bonferroni’s correction on *p*-values but chose to report only medium and larger size effects (|*r*| > 0.3), along with their 95% confidence intervals.

#### fMRI data

Preprocessing and analysis of imaging data was performed with the AFNI software package^47^. Preprocessing included slice-timing and motion correction, warping of brain volumes to standard Talairach space, Gaussian spatial blurring (6 mm FWHM), and signal percent scaling.

To assess the effects of interest on BOLD activity, a multiple-regression model was specified and estimated for each subject. The model consisted of a baseline portion (a second-order Legendre polynomial, accounting for slow signal drifts, plus 6 motion parameters), and a set of event-related regressors representing the expected BOLD response to the following classes of stimuli: (*a*) correctly-responded congruent trials, (*b*) correctly-responded incongruent trials, (*c*) commission error trials, (*d*) omission error trials. Each event was modeled as a mini boxcar starting at the time of appearance of the Stroop trial and ending at the time of the participant’s response, followed by convolution with a gamma function accounting for the BOLD hemodynamic properties. If a response was not issued within the allotted 2-second window, the trial was marked as an omission error and the duration of the corresponding boxcar set at 2 s. We also included two gamma function-convolved regressors modeling the event-related response to the visual cue presented just before each task block and instructing to perform the task either “with maximum effort”, or “as relaxed as possible”. All the regressors were specified run-wise, that is, separate regressors were included for each run.

Group analyses were performed using one-sample *t*-tests on the subject-level contrast images corresponding to the effects of interest, with the ensuing *t*-maps converted to the *Z*-statistics. Unless otherwise indicated, these group-level *Z*-maps were thresholded using a combination of single-voxel significance level of *p* < 0.001, and a minimum cluster size determined by a permutation test at the group level to keep whole-brain false positive rates below 0.05^48^. This procedure, implemented in the AFNI routine 3dttest + + , was developed to address recent concerns about the lack of proper control of false positive rates in neuroimaging research^49^. We also assessed the presence of areas jointly sensitive to the effects of endogenous (*i.e*. EXR-RLX) and exogenous (*i.e*. incongr-congr) effort modulation, by computing the intersection of the corresponding thresholded statistical maps (separately for negative and positive activations). A similar procedure was followed to obtain a map of the areas jointly sensitive to the effect of endogenous effort modulation on the BOLD response to the effort-instruction cue, on one side, and to the actual task-block execution, on the other.

## Results

### Behavioural data

**Figure 2:**
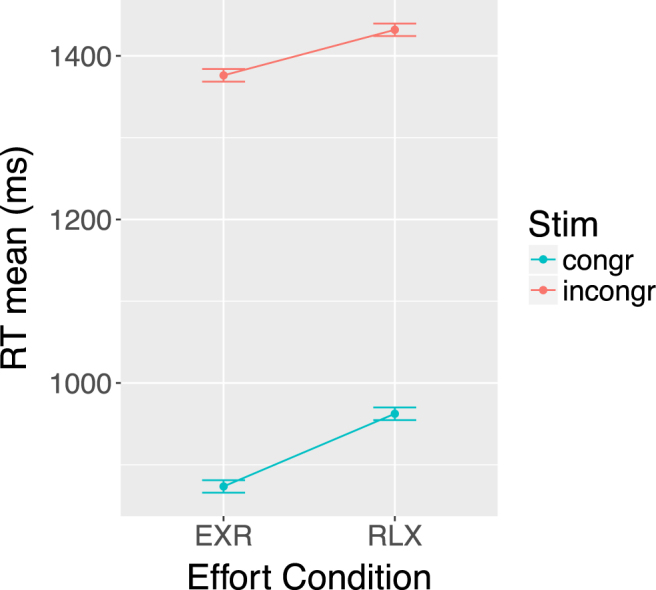
Effect of volitional effort (EXR, RLX) and stimulus type (congr, incongr) on response times (RT). In this and subsequent repeated-measures interaction plots, error bars display Fisher’s Least Significant Difference.

We observed a highly significant main effect of endogenous effort modulation on RTs for correct trials (EXR-RLX: *F*(1,44) = 30.30, *p* = 1.79e-06), in addition to the expected main effect of stimulus type (incongr-congr: *F*(1,44) = 1437.20, *p* = 3.07e-35). The *cond* × *stim* interaction was significant as well (*F*(1,44) = 9.44, *p* < 0.0036), showing a greater effect of endogenous effort recruitment for congruent than for incongruent trials (Table 1 and Fig. 2). Post-hoc Bonferroni-corrected *t*-tests revealed significantly faster responses in the EXR compared to the RLX condition, for both congruent (*p* = 1.59e-7) and incongruent (*p* = 0.00081) trials.

**Table 1:** Response times (RT, in ms; mean ± SE) for each *stim* × *cond* combination. Bonferroni-corrected *p*-values associated with EXR-RLX differences are shown in the last column. The numbers in parentheses in the second table section represent percentages of error trials with respect to the total number of trials in each cell. Note that the large size of the Stroop effect (incongr-congr) is a consequence of the particular version of the task we used, and is consistent with previously reported data^96^.

RT mean for correct trials	RLX	EXR	*p*(EXR-RLX)
congruent	962.4 ± 20.0	873.5 ± 17.3	<0.0001
incongruent	1431.9 ± 16.5	1376.2 ± 16.6	0.00081
**Number of errors per run**			
congruent	0.089 ± 0.029 (0.37%)	0.10 ± 0.034 (0.42%)	1.00
incongruent	3.27 ± 0.36 (13.6%)	2.81 ± 0.34 (11.7%)	0.11

We analyzed the number of errors per run with a 2 × 2 × 2 repeated-measures ANOVA, with *cond*, *stim* and error type as factors. In addition to the expected large effect of *stim* (*F*(1,44) = 83.29, p = 1.04e-11), we observed a marginally significant *stim* × *cond* interaction (*F*(1,44) = 4.22, *p* = 0.046); no other effect was significant. Post-hoc, Bonferroni-corrected t-tests revealed that the endogenous effort manipulation did not cause a significant change in the number of errors for either the congruent or the incongruent trials (see Table 1).

**Figure 3:**
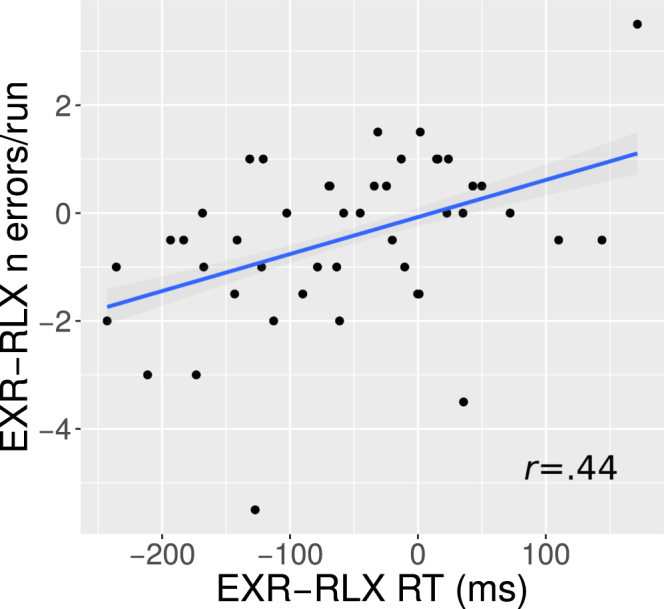
Correlation of individual EXR-RLX changes in response times (RT) and number of errors per run, for incongruent trials only. The value of Pearson’s correlation coefficient (*r*) is indicated in the plot.

Finally, we tested for a potential effort-related response speed-accuracy trade-off with a correlation analysis between the individual EXR-RLX changes in response times and the corresponding changes in the number of errors. The analysis was restricted to incongruent trials only, as the number of errors in the congruent trials was too small for meaningful statistics. The correlation was significant, of a medium-large effect size and *positive* (*r* = 0.44, *p* = 0.0027, 95%CI = [0.16,0.65]; Fig. 3).

### Subjective ratings

**Figure 4:**
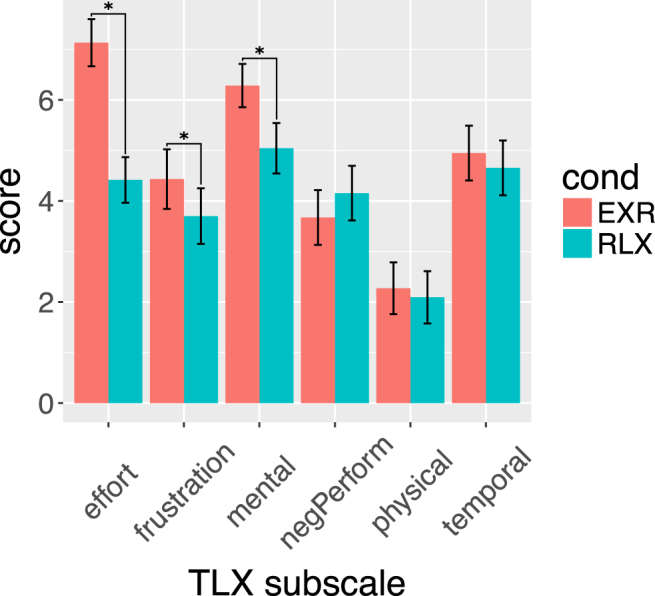
Subjective task-load ratings (NASA-TLX instrument) for the EXR and RLX conditions. The Performance subscale has been relabeled as ‘negPerform’, to remind the reader that its scores are high for ratings of poor performance. Error bars represent 95% confidence intervals for the mean scores. Paired comparisons marked with an asterisk are significant at a two-tail *α* < 0.05, Bonferroni-corrected.

Using the NASA-TLX psychometric instrument, EXR runs were rated significantly higher than RLX runs for what concerned the subjectively-felt amount of engaged effort (*Effort*), burden imposed on mental resources (*Mental Demand*), and aversiveness (*Frustration Level*); there was also a slight effect of endogenous effort modulation on temporal perception (*Temporal Demand*), whereby the subjectively-felt time pressure of the task was marginally lower in the RLX compared to the EXR condition (Fig. 4). The remaining NASA-TLX subscores were not significantly different between the EXR and the RLX conditions (Supplementary Table S1).

### Heart rate

**Figure 5:**
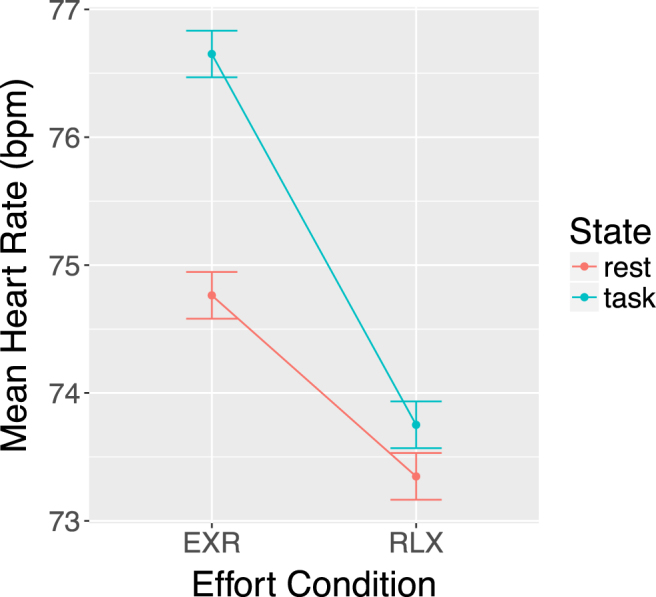
Average heart rate during task and rest blocks in the two endogenous effort modulation conditions.

Cardiac frequency was significantly affected (Fig. 5) by endogenous effort modulation (EXR-RLX: *F*(1,44) = 51.28, *p* = 6.64e-9), work state (task-rest: *F*(1,44) = 14.67, *p* = 0.0004), and their interaction (*cond* × *state*: *F*(1,44) = 33.52, *p* = 6.87e-7). Post-hoc Bonferroni-corrected t-tests showed that, while HR increased significantly in the EXR condition compared to the RLX condition during both rest (ΔHR = 1.42 bpm, *p* = 5.6e-6) and task blocks (ΔHR = 2.90 bpm, *p* = 1.7e-8), the effect was larger for task blocks. This was expected, as the instruction to engage either maximum effort or try to stay as relaxed as possible was displayed just before the task blocks, and in fact referred specifically to task execution.

### Correlations between EXR-RLX changes in performance, heart rate, and subjective ratings

**Figure 6:**
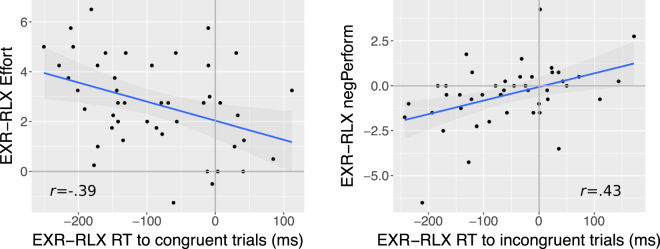
Objective performance *v.s*. subjective experience in the Stroop task. The left graph shows the across-subject correlation between EXR-RLX differences in response times (RT) to congruent trials and EXR-RLX differences in the TLX-NASA *Effort* subscale. The right graph shows the correlation between EXR-RLX differences in response times (RT) to incongruent trials and and EXR-RLX differences in the TLX-NASA *negPerform* subscale. Pearson’s correlation coefficients are reported within the plot area.

We observed a medium-size negative correlation between EXR-RLX differences in *Effort* ratings and in RT for congruent (*r* = − 0.39, *p* = 0.0083, 95%CI = [−0.61,−0.11]; see Fig. 6, left side), but not incongruent trials (*r* = − 0.24, *p* = 0.11, 95%CI = [−0.50,0.055]). Also, EXR-RLX differences in *negPerform* ratings correlated significantly with the corresponding RT differences for incongruent trials (*r* = 0.43, *p* = 0.0032, 95%CI = [0.16,0.64]; see Fig. 6, right side), and marginally for congruent trials (*r* = 0.30, *p* = 0.044, 95%CI = [0.0096,0.55]).

No significant across-subjects correlations were observed between EXR-RLX differences in mean HR during task performance and EXR-RLX differences in mean RT (for either congruent or incongruent trials), number of errors per run (incongruent trials only), or TLX-NASA ratings

### Neuroimaging data

#### Initial response to the endogenous-effort modulation cue

**Figure 7:**
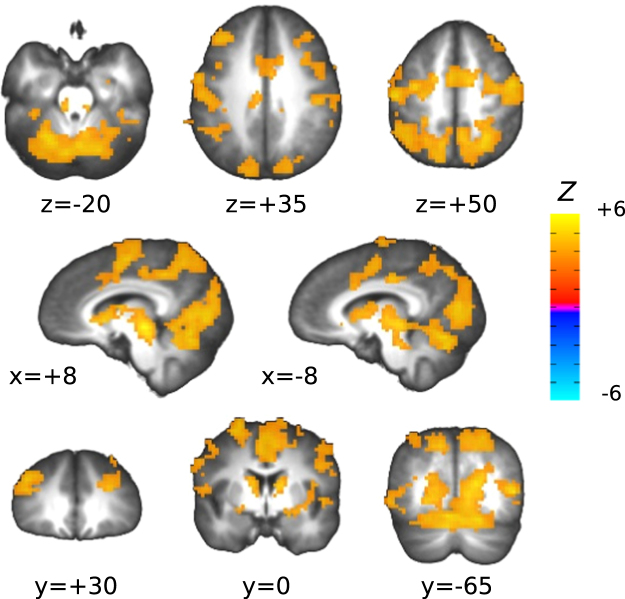
Activation map for the contrast cueEXR-cueRLX. Top, middle, and bottom rows illustrate representative slices in axial, sagittal, and coronal view, respectively. In this and other brain maps, the underlying anatomy is the average of the Talairach-warped anatomical volumes of all participants.

A whole-brain analysis testing for differences in the amplitude of the BOLD response to the instruction cue for endogenous effort modulation appearing immediately before each task block (contrast: cueEXR-cueRLX), showed a large, widespread activation pattern (Fig. 7), with its global peak in the brain stem (Talairach coordinates: 8, −22, −4; see Supplementary Fig. S1 for greater detail). Activated areas included the fronto-parietal attention system (superior parietal cortex, supplementary and pre-supplementary motor area, frontal eye fields and superior frontal gyrus, dorsolateral prefrontal cortex), regions associated with saliency, evaluation and sympathetic outflow (anterior/middle cingulate gyrus, BA9, insula, caudate head), occipital and posterior temporal cortices, thalamus and cerebellum (we do not report a table of activated clusters, as most of the above activations coalesced into a single cluster of around 10000 voxels, at the chosen statistical threshold of cluster-level false positives rate *α* < 0.05).

#### Relationship between effort-related changes in HR and in the cue-related BOLD response

**Figure 8:**
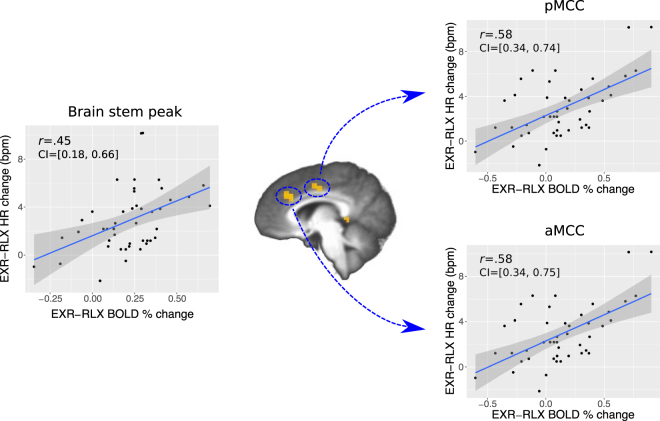
Relationship between individual EXR-RLX changes in heart rate (HR) and in the BOLD response to the effort cue. On the left side, the HR changes are regressed onto the BOLD average contrast of parameter estimates in a 3mm-radius sphere centered on the global activation peak from the cueEXR-cueRLX contrast (Talairach coordinates: 8, −22, −8). On the right side, similar graphs are displayed for the anterior and posterior midcingulate clusters (aMCC, pMCC) from the whole-brain analysis. Pearson’s correlation coefficients (*r*) and 95% confidence intervals (CI) are indicated in the plots.

An across-subjects correlation analysis was conducted between the EXR-RLX changes in HR and in the BOLD response to the visual cue indicating the prescribed effort investment for the upcoming task block. We first performed a targeted analysis, focusing on the global peak of the cueEXR-cueRLX activation map, located in the brain stem. More specifically, we extracted the average BOLD contrast value from a 3mm-radius sphere centered on the peak, for every subject, and correlated the individual values with the corresponding EXR-RLX changes in HR during the task blocks. The analysis showed a significant positive correlation with a medium-large effect size (Fig. 8, left side). Then, in order to examine the potential involvement of other brain regions in the same effect, we also extended the correlation analysis to the whole brain: we observed a number of significant clusters in various regions (Table 2 and Supplementary Fig. S2), including the midcingulate cortex (Fig. 8, right side), although the original brain stem activation did not survive the whole-brain corrected significance threshold.

**Table 2:** Activated clusters for the whole-brain correlation analysis between the individual magnitude of the EXR-RLX BOLD response to the cue preceding the task blocks and the corresponding EXR-RLX differences in heart rate. Size is reported in voxels (3 × 3 × 3 mm), and both Talairach coordinates and statistical *Z*-values refer to cluster peaks. Abbreviations: R = right, L = left, RL = bilateral.

Region	Size	x	y	z	Z-value
R Superior Frontal Gyrus	81	22.5	34.5	38.5	4.21
L Precentral Gyrus	79	−25.5	−28.5	71.5	4.63
L Fusiform Gyrus	76	−31.5	−49.5	−12.5	4.17
R Lingual Gyrus	64	7.5	−40.5	2.5	4.38
L Superior Occipital Gyrus	60	−10.5	−88.5	35.5	5.13
RL Posterior Midcingulate Cortex	55	1.5	−4.5	41.5	4.16
R Cingulate Sulcus Marginal Segment	53	13.5	−37.5	41.5	4.24
R Superior Parietal Gyrus	51	19.5	−64.5	62.5	4.45
R Anterior Midcingulate Cortex	42	7.5	25.5	32.5	4.23

#### Effect of endogenous effort engagement during task performance

**Figure 9:**
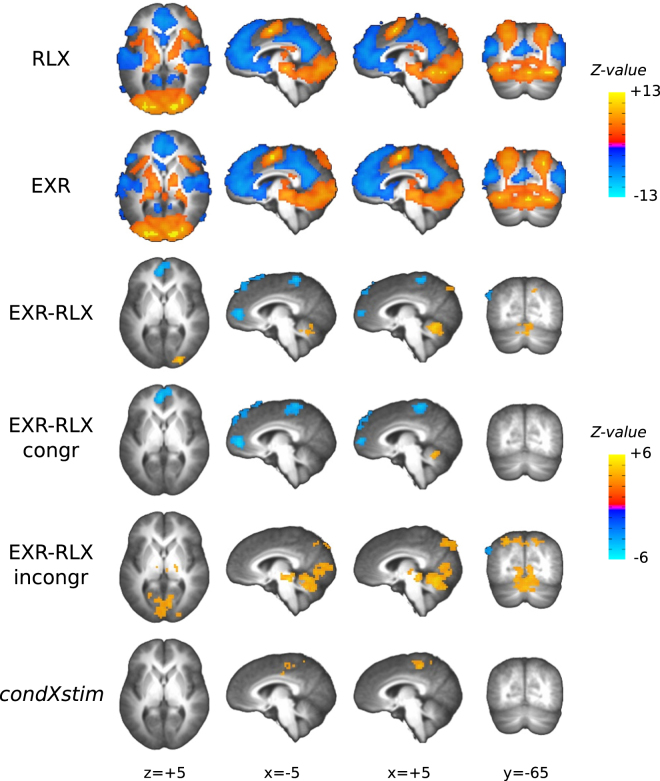
Statistical parametric maps for different contrasts. The top two rows illustrate task-related activation, compared to passive fixation, in the EXR and RLX conditions; the third row from top illustrates the main effect of endogenous effort modulation; the fourth and fifth row split the latter effect by stimulus type; finally, the bottom row shows the interaction of voluntary effort engagement and stimulus type, as identified by the [(EXR-RLX)/incongr −(EXR-RLX)/congr] contrast. Note that the top colourbar refers to the first two rows from top, and the bottom colourbar to the remaining four rows.

Performing the task blocks, compared to passive fixation, activated a set of regions implicated in externally-directed, attention-demanding processing, including anterior and posterior middle cingulate (aMCC, pMCC), supplementary and pre-supplementary motor area (SMA/preSMA), frontal eye fields (FEF), anterior insula (aIns), posterior parietal cortex, visual cortex, thalamus, and cerebellum; a strong deactivation of the DMN, including the hippocampus, was also observed, along with the superior and middle temporal gyri, and the paracentral lobule (Fig. 9, top two rows).

**Figure 10:**
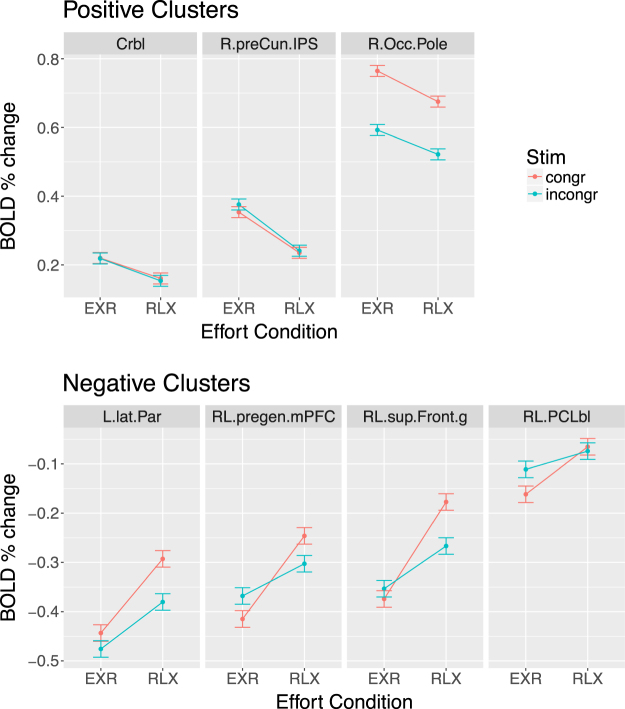
Average BOLD response to correct trials, split by level of endogenous effort and trial type, in each of the clusters from the EXR-RLX *Z*-map. Abbreviations: R = right, L = left, RL = bilateral.

Voluntary engagement of maximum effort, compared to relaxed task execution (contrast EXR-RLX), was overall associated with increased activity in the cerebellum (vermis and adjacent regions), occipital pole, posterior precuneus, as well as with a greater task-related decrease of activity in pregenual medial prefrontal cortex (mPFC), bilateral superior frontal gyrus, paracentral lobule and left lateral parietal cortex (Fig. 9, 3rd row, and Table 3). This effect, however, was not homogeneous across stimulus types, as can be gleaned from the 4th and 5th rows of Fig. 9, and from the cluster-averaged estimates of the BOLD response in Fig. 10. For congruent trials, voluntary investment of maximum exertion was associated with a stronger activation of the cerebellum, and a stronger deactivation of the medial prefrontal, superior frontal, and left lateral parietal components of the DMN, and of the paracentral lobule (Fig. 9, 4th row). For incongruent trials, voluntary investment of maximum exertion was associated with a stronger activation of the cerebellum as well, along with a stronger activation of brain stem structures, occipital and posterior parietal cortices (Fig. 9, 5th row). In order to verify that potential practice or fatigue effects were not biasing these findings, we also performed an additional analysis comparing the EXR-RLX effect in the first (runs 3–4) and in the second half (runs 5–6) of the scanning period included in the analyses: no significant clusters at the *α* < 0.05 level were detected.

**Table 3:** Significant clusters for the EXR-RLX contrast. Size is reported in voxels (3 × 3 × 3 mm), and both Talairach coordinates and statistical *Z*-values refer to cluster peaks. The cluster-averaged BOLD signal percent signal change is reported in the last column (Δ%). Abbreviations: R = right, L = left, RL = bilateral.

Region	Abbreviation	Size	x	y	z	Z-value	Δ%
***Positive Activations***							
Cerebellum	Crbl	245	4.5	−55.5	−12.5	5.02	0.062
R Occipital Pole	R.Occ.Pole	79	25.5	−85.5	5.5	4.98	0.080
R Precuneus and Intraparietal Sulcus	R.preCun.IPS	51	7.5	−73.5	47.5	3.96	0.126
***Negative Activations***							
RL Superior Frontal Gyrus	RL.sup.Front.g	242	−10.5	34.5	56.5	−4.81	−0.142
RL Pregenual Medial Prefrontal Cortex	RL.pregen.mPFC	146	−4.5	46.5	11.5	−4.74	−0.117
RL Paracentral Lobule	RL.PCLbl	104	−4.5	−37.5	59.5	−4.85	−0.067
L Lateral Parietal cortex	L.lat.Par	63	−49.5	−73.5	38.5	−4.47	−0.123

**Figure 11:**
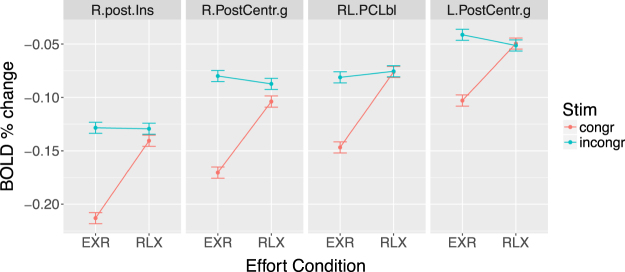
Interaction of voluntary effort investment and trial type on the BOLD response to correct trials in each of the clusters from the *cond* × *stim* *Z*-map.

A direct assessment of the *cond* × *stim* interaction identified significant clusters of BOLD signal change in the paracentral lobule, medial prefrontal cortex, right posterior insula, and cuneus (Fig. 9, 6th row, and Table 4); cluster-averaged estimates of the BOLD response are displayed in Fig. 11.

**Table 4:** Significant clusters for the cond × stim contrast. Size is reported in voxels (3 × 3 × 3 mm), and both Talairach coordinates and statistical *Z*-values refer to cluster peaks. The cluster-averaged BOLD signal percent signal change is reported in the last column (Δ%). Abbreviations: R = right, L = left, RL = bilateral.

Region	Abbreviation	Size	x	y	z	Z-value	Δ%
RL Paracentral Lobule	RL.PCLbl	138	−7.5	−22.5	56.5	4.56	0.065
L Postcentral Gyrus	L.PostCentr.g	83	−7.5	−49.5	59.5	4.13	0.063
R Postcentral Gyrus	R.PostCentr.g	63	34.5	−25.5	41.5	4.41	0.074
R Posterior Insula	R.post.Ins	38	37.5	−16.5	17.5	3.98	0.073

**Figure 12:**
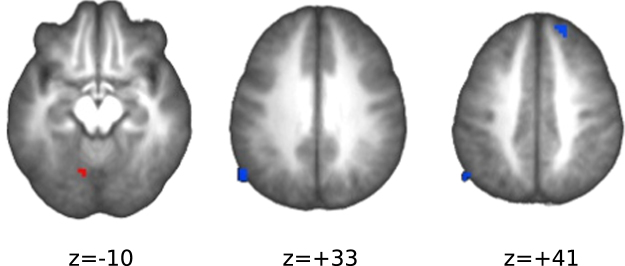
Areas jointly sensitive to exogenous and endogenous effort modulation. The map was obtained by a binary intersection of the thresholded statistical maps corresponding to the two effects. Red indicates joint positive activation, blue joint negative activation.

The identification of areas jointly sensitive to endogenous (EXR-RLX) and exogenous (incongr-congr) effort modulation, revealed shared positive activation in the left lingual/fusiform gyrus, and shared deactivation in the DMN nodes of left lateral parietal cortex and right superior frontal gyrus (Fig. 12 and Supplementary Table S2).

**Figure 13:**
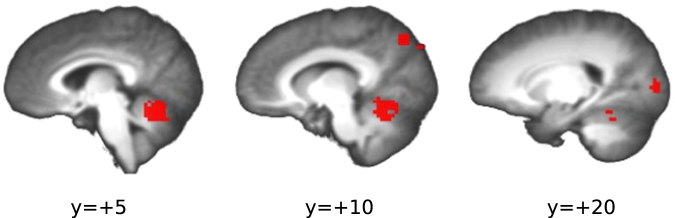
Areas jointly sensitive to EXR-RLX effects during the preparatory cue and during the actual task execution. The map was obtained by a binary intersection of the thresholded statistical maps corresponding to the two effects. All clusters correspond to positive activations in the original EXR-RLX contrasts.

Finally, areas sensitive to the EXR-RLX effect during both the preparatory visual cue and the actual task execution, comprised positive-only shared activations in the cerebellum (bilateral, but more extended in the right hemisphere), right occipital pole and right precuneus (Fig. 13 and Supplementary Table S3). We also investigated whether there was a significant relationship between the magnitude of the EXR-RLX BOLD changes in response to the task cue and during the task itself in the ROIs identified by the EXR-RLX task contrast. The only two significant correlations we observed (*p*-value uncorrected for the number of ROIs, but both with a medium effect size) were for incongruent trials in the cerebellar ROI (*r* = 0.37, *p* = 0.014, 95%CI = [0.080,0.60]), and in the right precuneus/IPS ROI (*r* = 0.30, *p* = 0.042, 95%CI = [0.01,0.55]).

### Relationship between effort-related changes in BOLD and behavior

**Figure 14:**
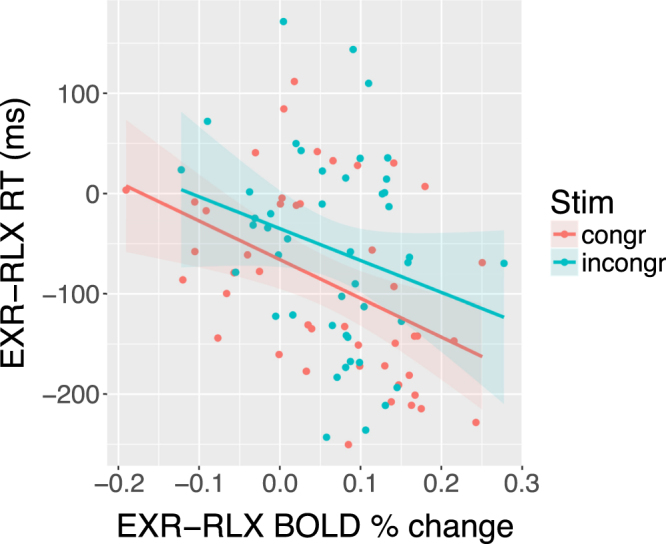
Across-subjects EXR-RLX changes in RT *vs*. EXR-RLX changes in cerebellar BOLD activation. The displayed linear relationship was significant at a Bonferroni-corrected *p* = 0.025.

We tested for a linear relationship between the individual average changes in response times associated with voluntary effort modulation, and the corresponding changes in brain activation. For each of the clusters from the EXR-RLX contrast, the average contrasts of parameter estimates for the BOLD response were computed, separately for congruent and incongruent stimuli; an ANCOVA with separate slopes was then set up and estimated, with the above computed contrast values, stimulus type and their interaction as predictors, and RT as the dependent variable. After applying Bonferroni’s correction for the number of examined clusters (*n* = 7), only the cerebellum displayed a significant relationship between EXR-RLX changes in brain activation and in RT (*p* = 0.025; Fig. 14, left side); no region displayed a significant modulation of the effect by stimulus type.

**Figure 15:**
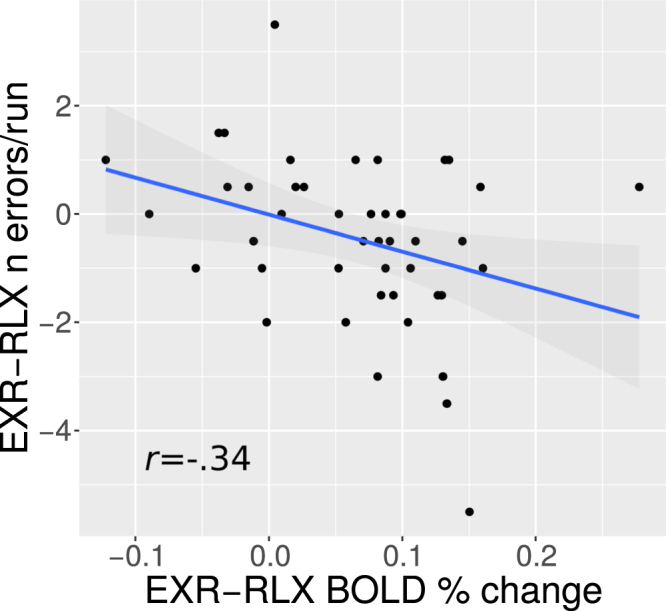
Correlation of individual EXR-RLX BOLD changes in the cerebellar cluster and in the number of errors per run, for incongruent trials only. The value of Pearson’s correlation coefficient (*r*) is indicated in the plot.

We also ran a correlation analysis between the average EXR-RLX BOLD differences in the ROIs from the EXR-RLX contrast and the corresponding changes in number of errors per run. The analysis was restricted to incongruent trials only, as the number of errors in the congruent trials was too low for meaningful statistics. The only ROI with a significant effect was again the one in the cerebellum, showing a negative correlation of medium size (*r* = −0.34, *p* = 0.024, 95%CI = [−0.57,−0.047]; Fig. 15).

## Discussion

This study was aimed at investigating a specific aspect of effortful mental activity that has not received much attention in the scientific literature yet, namely the voluntary modulation of invested effort in the execution of a demanding task. Participants were asked to perform a particularly challenging version of the Stroop task, either “with maximum effort”, or “as relaxed as possible”. The instructions were kept deliberately simple because we were interested in the effects of common exhortations to “put more effort” into a task or “to just relax” into it, so frequently employed in daily life. We chose to mimic the form of such sentences in the experimental instructions, in order to maintain their ecological validity. Subjective ratings collected at the end of each task run confirmed that the instruction to engage maximum effort was associated with higher scores for effort, mental demand, and aversive feeling.

Behaviourally, the voluntary engagement of maximum exertion resulted in faster response times, compared to performing the task in a relaxed fashion, with the effect being significantly stronger for the congruent (easier) trials. The number of errors did not differ significantly between the two conditions, although the overall low error rates may have precluded the detection of such an effect. We did not find evidence for an effort-related trade-off mechanism between response speed and accuracy. Quite to the contrary, we observed a significant *positive* correlation between EXR-RLX changes in individual response times and number of errors on incongruent trials showing that, on average, the individuals who increased their response speed more going from the RLX to the EXR condition were also the individuals who *decreased* their number of errors more going from the RLX to the EXR condition (Fig. 3).

We did observe some statistically significant associations between objective task-performance and subjective experience, which may support a role of behavioural monitoring in informing first-person phenomenology. Subjects with larger increases in response speed to congruent trials in the EXR compared to the RLX condition, tended to report larger increases in the subjectively-judged amount of invested effort (Fig. 6, left). Subjects with larger increases in response speed to incongruent trials in the EXR compared to the RLX condition, tended to subjectively assign better ratings to their task performance (Fig. 6, right). On the other hand, since EXR-RLX changes in HR and in TLX-NASA ratings were not significantly correlated, we did not find sufficient evidence for a role of cardiac function interoception in mediating subjective feelings of various dimensions of task load, including effort-related constructs.

The observed performance changes when participants were asked to execute the task with maximum exertion may be due to a state of increased neural readiness, mediated by attentional, arousal and premotor circuits. The brain response to the visual cue that preceded each task block, instructing to perform the task either with maximum exertion or as relaxed as possible, showed indeed a marked difference between the two conditions (Fig. 7). Compared to relaxed performance, the cue for maximum exertion triggered an extensive activation pattern that included frontoparietal attentional circuits, SMA/preSMA, aMCC, antINS, occipital visual cortices, thalamus, caudate head, and cerebellum. Most of these regions can be considered part of an executive control and cognitive effort network^16,50,51^. The preSMA, which is contiguous to, and frequently coactivated with the dorsal ACC/MCC, has been implicated in the intention to act^52–54^. The aMCC itself has been hypothesized to be responsible for biasing action selection with a reinforcing affective tone, according to the available information about reward and error^55,56^, which may include a representation of the projected energy costs of competing action sets. Its activity has also been linked to the increase of sympathetic tone during effortful cognitive and motor tasks^57^, and impairment of both sympathetic output and feeling of effort has indeed been reported with lesions of the ACC/aMCC^58^. These regions receive extensive projections from the mesolimbic dopaminergic reward system and are in general tightly connected with the basal ganglia^59^, structures whose functional and structural deficits often underlie the phenomenon of central fatigue^60^.

Notably, the most significant voxels for the contrast cueEXR-cueRLX were located in a region of the brain stem comprising the red nucleus – and more generally the upper tract of the reticular formation – the locus coeruleus, the substantia nigra, and the superior colliculus. The red nucleus receives projections from motor and premotor cortical areas and from the FEFs, and relays cerebellar signals to the spine via the rubrospinal tract. In our case, its joint activation with the anterior cerebellum (lobules IV–VI), which is involved in preparation for upper limb articulatory movement^61,62^, fits well with the neural requirements of readying oneself to perform the upcoming visuomotor task with maximum exertion. Note also that the more widespread, bilateral cerebellar activation evoked by the preparatory cue, became mainly ipsilateral to the performing hand during the actual task execution (compare Fig. 7 with Fig. 9, 3rd row from top; see also Fig. 13), suggesting an activation sequence that included a generic preparatory phase (involving, *e.g*. the modulation of arousal-related postural subtle changes), followed by a more restricted and task-specific response. Other nuclei in the reticular formation have a critical role in maintaining arousal and in cardiovascular control, while the noradrenergic locus coeruleus is a key orchestrator of stress-related sympathetic responses. In our study, the increased activation of this region was in fact accompanied by an increase in HR during task execution (Fig. 5), and the EXR-RLX changes in the two measures were significantly correlated (Fig. 8), in agreement with previous reports of HR increases related to mental effort^63–65^. Furthermore, the examination of such correlation across the whole brain, revealed the significant involvement of other cortical regions and, notably, of the anterior and posterior midcingulate (Fig. 8). These areas, which project to both the reticular formation and the spinal cord, have been recently claimed to be the human homologues of the major sources of top-down cortical influence over the adrenal medulla^66^. The reticular formation is also implicated in the integration of visual, vestibular, and postural signals during states of heightened attention, such as those presumably triggered by the intention to engage maximum effort. Interestingly, volumetric changes of this structure have been associated with chronic fatigue syndrome^67,68^, a condition characterized by unusually elevated sensations of effort. The superior colliculus is involved in the generation of saccadic eye movements and in covert shifts of attention, processes activated by the visual search component of the Stroop task version we adopted. Finally, the substantia nigra has both a motivational and a motor control function, with complex connections to the other basal ganglia nuclei that involve both dopaminergic and GABAergic transmission. In short, the above findings suggest that the willed preparation to engage fully one’s own mental resources, in response to the instruction cue, entails the generation of an orchestrated activity that triggers a widespread cortical and subcortical response, determining a state of increased motivation, arousal, attention and visuomotor readiness^69,70^.

Concerning the main effect of voluntary exertion on BOLD activation related to the actual task execution (EXR-RLX contrast), rather than to its cue-triggered preparatory phase, a few considerations can be made. First, engagement of maximum effort tended to amplify both task-related positive activations (cerebellum, right precuneus/intraparietal sulcus, right occipital pole) *and* deactivations (DMN areas and paracentral lobule) (Fig. 10). This may reflect a broad, catecolamine-mediated increase in neural gain^71–73^, following the strong activation of the reticular formation, locus coeruleus and midcingulate at the presentation of the cue for maximum effort engagement (Fig. 7); however, the lack of a significant correlation between EXR-RLX changes in RT and HR, suggests that the former are unlikely to be driven solely by a generalized modulation of autonomic output. Second, all the areas exhibiting an amplification of positive task-related activation in the maximum exertion condition showed an anticipatory increase of BOLD signal already during the presentation of the visual cue for maximum exertion (Fig. 13). Third, incongruent trials caused a greater deactivation, compared to congruent trials, of key regions of the DMN–mPFC, lateral parietal cortex, and superior frontal gyrus–but *only* in the relaxed performance condition (Fig. 10, lower half). The voluntary engagement of maximum effort further increased general DMN deactivation, but more so for congruent trials, whose deactivation levels approached those of incongruent trials. In general, the degree of deactivation of the DMN during processing of exogenous stimuli is known to increase with the difficulty of the task^36^, a process believed to reflect the suppression of distracting, task-unrelated thoughts within a limited-resources cognitive economy^74^. Finally, while the significant involvement of the cerebellum in the voluntary modulation of invested effort observed here was not among our a priori hypotheses, its implication in the general premotor/attentional process of readying oneself up for the performance of the task is plausible. The cerebellum has been previously linked to effort- or fatigue-related processes, attention, and word-reading^75–78^, with an increasing appreciation of its role as a critical system at the interface of motor behavior and cognition^79–82^. Note that the activity of the cerebellum in the effects described in the present study is not consistent with a simple effort-related speeding up of motor responses to the detriment of accuracy: on average, individuals whose cerebellar activity increased more from the RLX to the EXR condition were also the individuals whose number of errors *decreased* more from RLX to EXR (Fig. 15).

Interestingly, also the brain regions characterized by a significant interaction of invested effort and trial type in the whole-brain analysis (Fig. 11) all displayed task-related *de*-activation; note also that (*a*) these areas are not part of the DMN, and (*b*) the maximum exertion condition, compared to relaxed performance, only amplified the deactivations related to *congruent* trials (Fig. 11). The anatomical location of these clusters–right posterior insula, medial superior parietal cortex, and middle portion of right postcentral gyrus–suggests a role in somatosensory processing. In particular, the medial superior parietal cluster and the right postcentral gyrus cluster are well aligned somatotopically with the cortical representations of the bilateral foot and of the left hand, respectively, *i.e*. the limbs not explicitly engaged by the task motor response (requiring subjects to use their right hand). In the relaxed performance condition, these regions were similarly deactivated, compared to passive fixation, by congruent and incongruent trials. The maximum effort condition, by contrast, strongly amplified deactivations for congruent trials only, having a modest or negligible effect on incongruent trials. A possible explanation is that the deactivation of this circuit reflects a mechanism of dampening somatosensory afferences from non-engaged limbs when engrossed in the performance of a demanding action^83^, similarly to the decrease of BOLD signal in the ipsilateral somatosensory cortex observed during both anticipation^84^ and delivery^85^ of sensory stimulation.

We propose the following tentative interpretation of the differential effects of voluntary effort investment on congruent and incongruent trials. When the execution of a task requires a substantial quota of the available cognitive resources, those left for maintaining online and implementing ancillary task-set instructions (such as those related to effort engagement, in our case) may be insufficient. In the present study, where incongruent trials were much more demanding than congruent trials, the effect of voluntary effort investment may thus be expected to manifest more clearly for congruent than incongruent trials. From a slightly different perspective, the *proactive* control process^86^ corresponding to the intention of engaging maximum effort, which is set up at the moment of the cue presentation, is then bound to be weakened considerably during the performance of the high-demand incongruent trials. This interpretation, which may appear counter-intuitive at first, is in fact consistent with a limited-capacity model of attention^17^. Indeed, the voluntary application of maximum exertion was associated with (*i*) a greater speeding up of motor responses for congruent than for incongruent trials, (*ii*) a larger deactivation drop in DMN areas from their RLX levels, for congruent compared to incongruent trials (Fig. 10, lower half) and, (*iii*) a stronger deactivation of somatosensory circuits related to the limbs unengaged by the task for congruent than for incongruent trials (Fig. 11).

Finally, we did observe a few brain regions that responded in similar fashion to increases of exogenous and endogenous effort, *i.e*. statistically significant in both the EXR-RLX and the incongr-congr contrasts (Fig. 12). These regions (Supplementary Table S2) included DMN network nodes, such as the lateral parietal cortex and the superior frontal gyrus, and a small cluster in the lingual/fusiform gyrus. This finding confirms the notion that DMN deactivation is a general component of externally-directed effortful tasks, and extends it to the case where attentional effort is voluntarily increased instead of being merely mandated by the task. The cluster in the left lingual/fusiform gyrus, on the other hand, may be specifically related to the lexical characteristics of the Stroop task^87^. Overall, however, these results suggest that willed and task-driven engagement of mental effort (endogenous and exogenous effort, here) may be quite distinct processes, with substantially different neural substrates.

### Limitations

It is important to note that the present study investigated the effects of the *instruction* to willfully modulate the degree of invested effort and, while this produced observable effects in behavioural, subjective, autonomic, and brain data, other third-person measurements of the actual degree of invested effort, beyond HR data, were not collected. Although effort is a complex construct, with psychological and physiological interacting aspects that make a definitive objective assessment problematic, future studies may consider collecting additional measures such as pupillary diameter^88,89^, peripheral arterial tone^90^ and arterial blood pressure^42,91^, heart rate variability^64^, or skin conductance^65^, which have all been associated with changes in task load (note, however, that Fechir *et al*.^65^ reported similar changes in HR and skin conductance during a Stroop task).

Also, our study did not include a condition of “natural” performance, whereby subjects would be told simply to perform the task, without any explicit indication about voluntary effort investment. A legitimate question is then whether natural performance would be more similar to the EXR or to the RLX condition, or lie roughly half-way between the two. A more comprehensive investigation (*e.g*. using a larger sample and a cross-sectional design, in order to avoid carry-over effects) could add natural performance to the EXR and RLX conditions, and additionally examine how individual differences and personality traits contribute to the capacity to wilfully deviate from the natural setpoint.

Finally, given the strong impact of task demands on the effective maintenance of the effort-related intentions during task performance, caution should be exercised in generalizing the present findings to different tasks or even to different versions of the Stroop task. Furthermore, as our study was focused solely on the voluntary modulation of effort, additional research will be needed in order to directly compare volition-based and incentive-based engagement of effort, as such processes may involve at least partially distinct neural circuits and mechanisms.

### Concluding remarks

Beyond its importance in the cognitive science of executive function, the comprehension of effort-related processes is also extremely relevant in the clinic, as fatigue or loss of energy–with its dramatic impact on motivated behaviour–is one of the most common symptoms across psychiatric ilnesses^70,92^, and one that is particularly resistant to treatment. Outside of the clinical scope, the capacity of voluntary effort investment and its cognate construct of self-control, has been shown to predict success in both academic and work environments^93–95^. We hope that the present work contributes a valuable addition to this research field.

## Electronic supplementary material

Supplementary Information

## References

[1] Paas F, Tuovinen J, van Merrienboer J, Aubteen Darabi A (2005). A motivational perspective on the relation between mental effort and performance: Optimizing learner involvement in instruction. Educational Technology Research and Development.

[2] Blair C, Razza RP (2007). Relating effortful control, executive function, and false belief understanding to emerging math and literacy ability in kindergarten. Child Dev.

[3] Rothbart MK, Ahadi SA, Evans DE (2000). Temperament and personality: origins and outcomes. J Pers Soc Psychol.

[4] Hartlage S, Alloy LB, Vazquez C, Dykman B (1993). Automatic and effortful processing in depression. Psychol Bull.

[5] Chaudhuri A, Behan PO (2004). Fatigue in neurological disorders. Lancet.

[6] Sarter M, Gehring WJ, Kozak R (2006). More attention must be paid: the neurobiology of attentional effort. Brain Res Rev.

[7] Gilbert SJ, Burgess PW (2008). Executive function. Curr Biol.

[8] Botvinick MM, Rosen ZB (2009). Anticipation of cognitive demand during decision-making. Psychol Res.

[9] Croxson PL, Walton ME, O’Reilly JX, Behrens TEJ, Rushworth MFS (2009). Effort-based cost-benefit valuation and the human brain. J Neurosci.

[10] Kurniawan, I. T., Guitart-Masip, M. & Dolan, R. J. Dopamine and effort-based decision making. *Frontiers in Neuroscience* **5**10.3389/fnins.2011.00081 (2011). 10.3389/fnins.2011.00081 PMC3122071 21734862

[11] Kurzban R, Duckworth A, Kable JW, Myers J (2013). An opportunity cost model of subjective effort and task performance. Behavioral and Brain Sciences.

[12] Westbrook A, Braver TS (2015). Cognitive effort: A neuroeconomic approach. Cognitive, affective & behavioral neuroscience.

[13] de Croock MBM, van Merrienboer JJG, Paas FGWC (1998). High versus low contextual interference in simulation-based training of troubleshooting skills: effects on transfer performance and invested mental effort. Computers in Human Behavior.

[14] Camp G, Paas F, Rikers R, van Merrienboer J (2001). Dynamic problem selection in air traffic control training: a comparison between performance, mental effort and mental efficiency. Computers in Human Behavior.

[15] Salden RJ, Paas F, Broers NJ, van Merrienboer JJ (2004). Mental effort and performance as determinants for the dynamic selection of learning tasks in air traffic control training. Instructional Science.

[16] Shenhav A (2017). Toward a rational and mechanistic account of mental effort. Annual review of neuroscience.

[17] Kahneman, D. *Attention and Effort* (Prentice-Hall, Englewood Cliffs, 1973).

[18] Dehaene S, Kerszberg M, Changeux JP (1998). A neuronal model of a global workspace in effortful cognitive tasks. Proc Natl Acad Sci USA.

[19] Cauda F (2013). Functional anatomy of cortical areas characterized by von economo neurons. Brain Struct Funct.

[20] Paus T, Koski L, Caramanos Z, Westbury C (1998). Regional differences in the effects of task difficulty and motor output on blood flow response in the human anterior cingulate cortex: a review of 107 PET activation studies. Neuroreport.

[21] Engström M, Karlsson T, Landtblom A-M, Craig ADB (2015). Evidence of conjoint activation of the anterior insular and cingulate cortices during effortful tasks. Front Hum Neurosci.

[22] Gehring WJ, Goss B, Coles MG, Meyer DE, Donchin E (1993). A neural system for error detection and compensation. Psychological Science.

[23] Falkenstein M, Hoormann J, Christ S, Hohnsbein J (2000). Erp components on reaction errors and their functional significance: a tutorial. Biol Psychol.

[24] Carter CS (1998). Anterior cingulate cortex, error detection, and the online monitoring of performance. Science.

[25] Botvinick MM, Cohen JD, Carter CS (2004). Conflict monitoring and anterior cingulate cortex: an update. Trends Cogn Sci.

[26] Kerns JG (2004). Anterior cingulate conflict monitoring and adjustments in control. Science.

[27] Walton ME, Bannerman DM, Alterescu K, Rushworth MFS (2003). Functional specialization within medial frontal cortex of the anterior cingulate for evaluating effort-related decisions. J Neurosci.

[28] Mulert C, Menzinger E, Leicht G, Pogarell O, Hegerl U (2005). Evidence for a close relationship between conscious effort and anterior cingulate cortex activity. Int J Psychophysiol.

[29] Mulert C (2008). Single-trial coupling of eeg and fmri reveals the involvement of early anterior cingulate cortex activation in effortful decision making. Neuroimage.

[30] Fellows LK, Farah MJ (2005). Is anterior cingulate cortex necessary for cognitive control?. Brain.

[31] Mansouri FA, Tanaka K, Buckley MJ (2009). Conflict-induced behavioural adjustment: a clue to the executive functions of the prefrontal cortex. Nat Rev Neurosci.

[32] Vogt BA (2005). Pain and emotion interactions in subregions of the cingulate gyrus. Nat Rev Neurosci.

[33] Shulman G (1997). Common blood flow changes across visual tasks: I. increases in subcortical structures and cerebellum but not in nonvisual cortex. Journal of Cognitive Neuroscience.

[34] Raichle ME (2001). A default mode of brain function. Proc Natl Acad Sci USA.

[35] Binder JR (1999). Conceptual processing during the conscious resting state. A functional MRI study. J Cogn Neurosci.

[36] McKiernan KA, D’Angelo BR, Kaufman JN, Binder JR (2006). Interrupting the “stream of consciousness”: An fMRI investigation. Neuroimage.

[37] Mason MF (2007). Wandering minds: the default network and stimulus-independent thought. Science.

[38] Smallwood J, Fishman DJ, Schooler JW (2007). Counting the cost of an absent mind: mind wandering as an underrecognized influence on educational performance. Psychon Bull Rev.

[39] Smallwood J, Beach E, Schooler JW, Handy TC (2008). Going awol in the brain: Mind wandering reduces cortical analysis of external events. J Cogn Neurosci.

[40] Fox MD (2005). The human brain is intrinsically organized into dynamic, anticorrelated functional networks. Proc Natl Acad Sci USA.

[41] Oldfield RC (1971). The assessment and analysis of handedness: the Edinburgh inventory. Neuropsychologia.

[42] Gianaros PJ (2005). Anterior cingulate activity correlates with blood pressure during stress. Psychophysiology.

[43] Stroop JR (1935). Studies of interference in serial verbal reactions. Journal of experimental psychology.

[44] Peirce JW (2008). Generating stimuli for neuroscience using psychopy. Front Neuroinformatics.

[45] Hart, S. & Staveland, L. Development of nasa-tlx (task load index): Results of empirical and theoretical research. In Hancock, P. & Meshkati, N. (eds.) *Human mental workload*, 139–183 (North Holland, Amsterdam, 1988).

[46] Hart, S. G. Nasa-task load index (nasa-tlx); 20 years later. *Human Factors and Ergonomics Society Annual Meeting Proceedings* **50**, 904–908(5) (2006).

[47] Cox RW (1996). AFNI: software for analysis and visualization of functional magnetic resonance neuroimages. Comput Biomed Res.

[48] Cox, R. W., Reynolds, R. C. & Taylor, P. A. Afni and clustering: False positive rates redux. *bioRxiv*10.1101/065862 (2016).

[49] Eklund A, Nichols TE, Knutsson H (2016). Cluster failure: Why fmri inferences for spatial extent have inflated false-positive rates. Proc Natl Acad Sci USA.

[50] Dosenbach NUF, Fair DA, Cohen AL, Schlaggar BL, Petersen SE (2008). A dual-networks architecture of top-down control. Trends Cogn Sci.

[51] Power JD, Petersen SE (2013). Control-related systems in the human brain. Current opinion in neurobiology.

[52] Lau HC, Rogers RD, Ramnani N, Passingham RE (2004). Willed action and attention to the selection of action. Neuroimage.

[53] Lau HC, Rogers RD, Haggard P, Passingham RE (2004). Attention to intention. Science.

[54] Haggard P (2008). Human volition: towards a neuroscience of will. Nat Rev Neurosci.

[55] Gehring WJ, Willoughby AR (2002). The medial frontal cortex and the rapid processing of monetary gains and losses. Science.

[56] Walton ME, Devlin JT, Rushworth MFS (2004). Interactions between decision making and performance monitoring within prefrontal cortex. Nat Neurosci.

[57] Critchley HD (2003). Human cingulate cortex and autonomic control: converging neuroimaging and clinical evidence. Brain.

[58] Naccache L (2005). Effortless control: executive attention and conscious feeling of mental effort are dissociable. Neuropsychologia.

[59] Alexander GE, DeLong MR, Strick PL (1986). Parallel organization of functionally segregated circuits linking basal ganglia and cortex. Annu Rev Neurosci.

[60] Chaudhuri A, Behan PO (2000). Fatigue and basal ganglia. J Neurol Sci.

[61] Glickstein M, Sultan F, Voogd J (2011). Functional localization in the cerebellum. Cortex.

[62]  Stoodley CJ, Valera EM, Schmahmann JD (2012). Functional topography of the cerebellum for motor and cognitive tasks: an fmri study. Neuroimage.

[63] Gellatly IR, Meyer JP (1992). The effects of goal difficulty on physiological arousal, cognition, and task performance. The Journal of applied psychology.

[64] Capa RL, Audiffren M, Ragot S (2008). The interactive effect of achievement motivation and task difficulty on mental effort. Int J Psychophysiol.

[65] Fechir M (2010). Functional imaging of sympathetic activation during mental stress. Neuroimage.

[66] Dum RP, Levinthal DJ, Strick PL (2016). Motor, cognitive, and affective areas of the cerebral cortex influence the adrenal medulla. Proc Natl Acad Sci USA.

[67] Barnden LR, Kwiatek R, Crouch B, Burnet R, Del Fante P (2016). Autonomic correlations with mri are abnormal in the brainstem vasomotor centre in chronic fatigue syndrome. Neuroimage Clin.

[68] Barnden, L. R. *et al*. A brain mri study of chronic fatigue syndrome: evidence of brainstem dysfunction and altered homeostasis. *NMR Biomed*10.1002/nbm.1692 (2011). 10.1002/nbm.1692 PMC4369126 21560176

[69] Lütcke H, Gevensleben H, Albrecht B, Frahm J (2009). Brain networks involved in early versus late response anticipation and their relation to conflict processing. J Cogn Neurosci.

[70] Salamone JD, Yohn SE, López-Cruz L, San Miguel N, Correa M (2016). Activational and effort-related aspects of motivation: neural mechanisms and implications for psychopathology. Brain.

[71] Aston-Jones G, Cohen JD (2005). An integrative theory of locus coeruleus-norepinephrine function: adaptive gain and optimal performance. Annu Rev Neurosci.

[72] Warren CM (2016). Catecholamine-mediated increases in gain enhance the precision of cortical representations. The Journal of neuroscience: the official journal of the Society for Neuroscience.

[73] Eldar E, Cohen JD, Niv Y (2013). The effects of neural gain on attention and learning. Nature neuroscience.

[74] Smallwood J, Schooler JW (2015). The science of mind wandering: empirically navigating the stream of consciousness. Annual review of psychology.

[75] Massar SAA, Libedinsky C, Weiyan C, Huettel SA, Chee MWL (2015). Separate and overlapping brain areas encode subjective value during delay and effort discounting. NeuroImage.

[76] Persson J, Larsson A, Reuter-Lorenz PA (2013). Imaging fatigue of interference control reveals the neural basis of executive resource depletion. J Cogn Neurosci.

[77] Fiez JA, Petersen SE (1998). Neuroimaging studies of word reading. Proc Natl Acad Sci USA.

[78] Smallwood J (2013). The default modes of reading: modulation of posterior cingulate and medial prefrontal cortex connectivity associated with comprehension and task focus while reading. Front Hum Neurosci.

[79] Stoodley CJ (2012). The cerebellum and cognition: evidence from functional imaging studies. Cerebellum.

[80] Ramnani N (2012). Frontal lobe and posterior parietal contributions to the cortico-cerebellar system. Cerebellum.

[81] Peterburs J, Desmond JE (2016). The role of the human cerebellum in performance monitoring. Current opinion in neurobiology.

[82] Sokolov AA, Miall RC, Ivry RB (2017). The cerebellum: Adaptive prediction for movement and cognition. Trends in cognitive sciences.

[83] McGregor KM (2015). Reliability of negative bold in ipsilateral sensorimotor areas during unimanual task activity. Brain imaging and behavior.

[84] Porro, C. A. *et al*. Does anticipation of pain affect cortical nociceptive systems? *J Neurosci* **22**, 3206–3214 doi:20026310 (2002). 10.1523/JNEUROSCI.22-08-03206.2002 PMC6757517 11943821

[85] Mullinger KJ, Mayhew SD, Bagshaw AP, Bowtell R, Francis ST (2013). Poststimulus undershoots in cerebral blood flow and bold fmri responses are modulated by poststimulus neuronal activity. Proc Natl Acad Sci USA.

[86] Braver TS (2012). The variable nature of cognitive control: a dual mechanisms framework. Trends Cogn Sci.

[87] Fiebach CJ, Friederici AD, Müller K, von Cramon DY (2002). fmri evidence for dual routes to the mental lexicon in visual word recognition. Journal of cognitive neuroscience.

[88] Kahneman D, Beatty J (1966). Pupil diameter and load on memory. Science (New York, N.Y.).

[89] Beatty J (1982). Task-evoked pupillary responses, processing load, and the structure of processing resources. Psychological bulletin.

[90] Iani C, Gopher D, Lavie P (2004). Effects of task difficulty and invested mental effort on peripheral vasoconstriction. Psychophysiology.

[91] Wright RA, Stewart CC, Barnett BR (2008). Mental fatigue influence on effort-related cardiovascular response: extension across the regulatory (inhibitory)/non-regulatory performance dimension. International journal of psychophysiology: official journal of the International Organization of Psychophysiology.

[92] Demyttenaere K, De Fruyt J, Stahl SM (2005). The many faces of fatigue in major depressive disorder. The international journal of neuropsychopharmacology.

[93] Eigsti I-M (2006). Predicting cognitive control from preschool to late adolescence and young adulthood. Psychological science.

[94] Duckworth AL, Peterson C, Matthews MD, Kelly DR (2007). Grit: perseverance and passion for long-term goals. Journal of personality and social psychology.

[95] Duckworth AL (2011). The significance of self-control. Proc Natl Acad Sci USA.

[96] Gianaros PJ (2009). Heightened resting neural activity predicts exaggerated stressor-evoked blood pressure reactivity. Hypertension (Dallas, Tex.: 1979).

