# Supplementary material for: Voluntary modulation of mental effort investment: an fMRI study
Source: Sci Rep. 2017 Dec 8;7:17191. doi: 10.1038/s41598-017-17519-3 (PMC5722925; doi:10.1038/s41598-017-17519-3)
Supplement: Supplementary file 1 — Supplementary Information [file 41598_2017_17519_MOESM1_ESM.pdf]

Voluntary modulation of mental effort investment: an fMRI study

Omar T. Khachouf[1], Gang Chen[2], Davide Duzzi[1], Carlo A. Porro[1,3], and Giuseppe Pagnoni[1,3,*]

[1]Department of Biomedical, Metabolic and Neural Sciences, University of Modena and Reggio Emilia, Modena, 41125, Italy
[2]Scientific and Statistical Computing Core, National Institute of Mental Health, National Institutes of Health, Bethesda, MD, 20892, USA
[3]Center for Neuroscience and Neurotechnology, University of Modena and Reggio Emilia, Modena, 41125, Italy
[*]Corresponding author: giuseppe.pagnoni@unimore.it

September 21, 2017

Supplementary Information

NASA-TLX rating instrument

The NASA-TLX is a psychometric instrument assessing the subjective perception of task workload. It consists of six subscales, which were explained to the volunteers according to the following description:

Mental Demand How hard was the task mentally?

Physical Demand How hard was the task physically?

Temporal Demand How much time pressure did you feel due to the pace at which stimuli were presented?

Performance How successful do you think you were in performing the task correctly?

Effort How hard did you have to work to perform the task?

Frustration Level How insecure, discouraged, irritated, stressed and annoyed did the task make you feel?

Note that in the TLX instrument the Performance subscale is displayed graphically with high scores indicating *poor* performance: this is to maintain consistency with the other subscales (tasks with a higher mental workload are usually associated with poorer performance). In order to avoid confusion, in the figures and tables the subscale was relabeled as 'negPerform' (*i.e.* negative performance, where a high score indicates a subjective perception of *poor* performance). The mean `EXR-RLX` differences in the TLX scores, and their associated statistics, are reported in Table S1.

**TLX subscale**	**EXR-RLX**	$t(44)$	p**-value**
Mental Demand	1.24	6.27	$1.37 \times 10^{-7*}$
Physical Demand	0.18	1.30	0.20
Temporal Demand	0.29	2.09	0.042
negPerformance	-0.48	-1.88	0.067
Effort	2.72	9.97	$7.28 \times 10^{-13*}$
Frustration Level	0.73	3.24	0.0023^{*}

Table S1: Effect of endogenous effort modulation on various dimensions of perceived task workload (NASA-TLX instrument). The Performance subscale has been relabeled as 'negPerformance', to remind the reader that its scores are high for ratings of poor performance. Statistical values refer to a paired two-sample t-test, and are not Bonferroni-corrected. The tests marked with an asterisk remain significant at $\alpha < 0.005$ after Bonferroni correction.

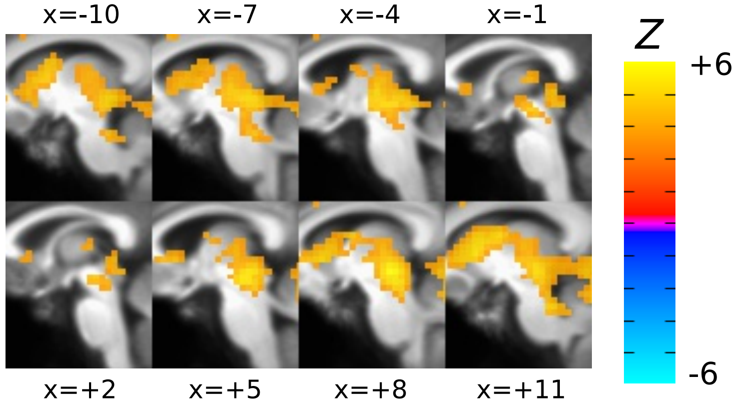

Figure S1: Activation map from the `cueEXR-cueRLX` contrast, where the brain stem activation is shown in greater detail.

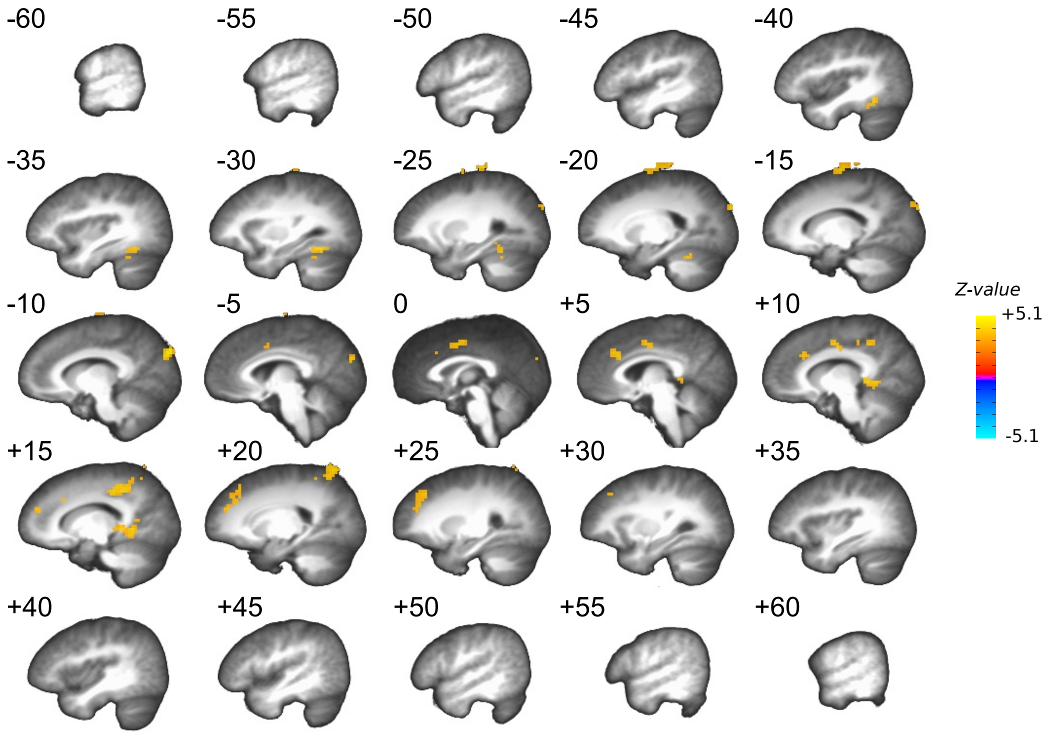

Figure S2: Activation map from the whole-brain correlation analysis betwen the individual `EXR-RLX` HR changes and the `cueEXR-cueRLX` BOLD response. The numbers reported in the top-left corner of each slice identify its x coordinate.

**Region**	**Size**	**x**	**y**	**z**
Positive Activations				
L lingual/fusiform gyr	3	-11.5	-62.5	-9.5
Negative Activations				
L lat parietal cortex	15	-53.7	-64.7	+35.5
R sup frontal gyr	14	+18.0	+41.8	+39.4
	2	+16.5	+36.0	+44.5
	2	+13.5	+36.0	+47.5

Table S2: Regions whose activity was significantly modulated by both exogenous and endogenous effort (*i.e.* contrasts `EXR-RLX` and `incongr-congr`). Size is reported in voxels ($3 \times 3 \times 3$ mm), and Talairach coordinates refer to the cluster center of mass. Abbreviations: L/R = left/right, lat = lateral, sup = superior, gyr = gyrus.

**Region**	**Size**	**x**	**y**	**z**
Cerebellum (mostly R)	198	+6.3	-56.7	-14.6
R occipital pole	30	+19.5	-86.4	+7.4
R precuneus	19	+10.3	-69.6	+45.1
	4	+9.0	-83.2	+37.8

Table S3: Regions whose activity significantly increased, in the `EXR` compared to the `RLX` condition, in response to both the preparatory visual cue for effort engagement and the actual Stroop task execution. Size is reported in voxels ($3 \times 3 \times 3$ mm), and Talairach coordinates refer to the cluster center of mass. Abbreviations: R = right.
